# CHANGES IN ADIPOSITY AND COGNITION AMONG EARLY POSTMENOPAUSAL WOMEN FOLLOWING HORMONE TREATMENT

**DOI:** 10.1093/geroni/igad104.2231

**Published:** 2023-12-21

**Authors:** Maritza Dowling, JoAnn Manson, Frederick Naftolin, Sherman Harman, Dustin Hammers, Marcelle Cedars, kejal Kantarci, Carey Gleason

**Affiliations:** George Washington University, Washington, District of COlumbia, United States; Brigham & Women’s Hospital/Harvard University, Boston, Massachusetts, United States; NYU School of Medicine, New York City, New York, United States; Phoenix VA Health Care System, Phoenix, Arizona, United States; Indiana University, Indianapolis, Indiana, United States; University of California, San Francisco, San Francisco, California, United States; Mayo Clinics, Rochester, Minnesota, United States; University of Wisconsin, Madison, Wisconsin, United States

## Abstract

Evidence suggests that early postmenopausal hormone treatment (MHT) may attenuate metabolic effects on dementia pathogenesis. Using Kronos Early Estrogen Prevention Study (KEEPS) and KEEPS-Continuation data, we investigated heterogeneity in the associations between longitudinal central adiposity (CA), MHT, and cognition, hypothesizing that CA would be related to cognition and that MHT would favorably alter this relationship. KEEPS participants (previously randomized to 48 months treatment with placebo or HT with oral conjugated equine estrogens + progesterone or transdermal 17-β-estradiol+ progesterone) were recruited for KEEPS-Continuation ~10 years post-randomization. Cognitive tests from both studies were analyzed as four-factor scores. Using the original KEEPS data (n=662), growth mixture modeling identified distinct CA trajectories (classes) across 48 months of MHT, which predicted cognition at 48 months and ~10 years, adjusting for covariates. Adiposity trajectories included “low-CA” and “high-CA” classes. Low-CA women exhibited stable low-CA throughout MHT. High-CA women’s adiposity was initially elevated but declined significantly after 48 months of MHT. After adjusting for covariates, the high (versus low) CA class predicted significantly lower cognitive factors scores at month 48 with one exception – Verbal-Learning & Memory. Ten years later, high-CA women (n=299) scored significantly lower on two factors. MHT predicted neither CA trajectories nor cognitive performance at 48 months or 10 years later. Adiposity levels significantly declined in the high-CA class on MHT but not placebo and was associated with diminished cognitive function. CA influenced short-and long-term cognition. Organ/tissue studies should examine how heterogeneity in adiposity levels interacts with MHT and other factors driving dementia pathogenesis.

